# Supplementary material for: Associations between Allergic and Autoimmune Diseases with Autism Spectrum Disorder and Attention-Deficit/Hyperactivity Disorder within Families: A Population-Based Cohort Study
Source: Int J Environ Res Public Health. 2022 Apr 8;19(8):4503. doi: 10.3390/ijerph19084503 (PMC9025211; doi:10.3390/ijerph19084503)
Supplement: Supplementary file 1 [file ijerph-19-04503-s001.zip › ijerph-1614536-supplementary.pdf]

Supplementary Table S1. Reference ICD9/10 code of allergic and autoimmune disease.

Allergic disease	ICD-9	ICD-10
Asthma	493	J45
Allergy rhinitis	477	J30
Atopic dermatitis	691	L20
Autoimmune disease	ICD-9	ICD-10
Rheumatoid arthritis	714	M05, M08, M45
Sjogren syndrome	710.2	M35.0
Psoriasis	696	L30.5, L40, L41, L42, L44
Systemic lupus erythematosus	710.0	M32
Ankylosing spondylitis	720.0	M45.9

Supplementary Table S2. Full sibling 's ASD, ADHD and immune diseases and allergic diseases.

Disease	N=14,576
Autism spectrum disorder	141 (1.0)
Attention-deficit/hyperactivity disorder	1270 (8.7)
Any allergic diseases	7905 (54.2)
Asthma	3999 (27.4)
Allergy rhinitis	5685 (39.0)
Atopic dermatitis	2968 (20.4)
Any autoimmune diseases	39 (0.3)
Rheumatoid arthritis	5 (0.003)
Sjogren syndrome	5 (0.003)
Psoriasis	26 (0.2)
Systemic lupus erythematosus	4 (0.003)
Ankylosing spondylitis	0 (0.0)

Supplementary Table S3. The association between common immune and allergic disorder of half-sibling and individual's ASD and ADHD using familiar aggregation.

	OR (95% CI)	P-value
Autism spectrum disorder		
Any autoimmune diseases	3.26 (0.45-23.81)	0.245
Rheumatoid arthritis	0.00 (0.00->99.99)	0.981
Sjogren syndrome	4.84 (0.65-35.87)	0.123
Psoriasis	0.00 (0.00->99.99)	0.932
Systemic lupus erythematosus	1.48 (1.00-2.17)	0.047
Any allergic diseases	1.55 (1.07-2.26)	0.021
Asthma	3.29 (0.45-24.20)	0.242
Allergy rhinitis	0.00 (0.00->99.99)	0.991
Atopic dermatitis	4.82 (0.65-35.69)	0.124
Attention-deficit/hyperactivity disorder		
Any autoimmune diseases	0.00 (0.00 ->99.99)	0.934
Rheumatoid arthritis	0.00 (0.00->99.99)	0.923
Sjogren syndrome	0.00 (0.00->99.99)	0.980
Psoriasis	0.00 (0.00->99.99)	0.951
Systemic lupus erythematosus	1.06 (0.94-1.21)	0.343
Any allergic diseases	1.16 (1.00-1.35)	0.043
Asthma	0.00 (0.00->99.99)	0.987
Allergy rhinitis	0.00 (0.00->99.99)	0.932
Atopic dermatitis	0.00 (0.00->99.99)	0.950
